# Sensory stimulation—A way of creating mutual relations in dementia care

**DOI:** 10.3402/qhw.v9.23888

**Published:** 2014-07-08

**Authors:** Else Lykkeslet, Eva Gjengedal, Torill Skrondal, May-Britt Storjord

**Affiliations:** 1 Faculty of Health and Social Care, Molde University College, Molde, Norway; 2 Department of Global Health and Primary Care, Bergen University, Bergen, Norway; 3 Clinic for Mental Health, Moere and Romsdal Health Thrust, Molde, Norway

**Keywords:** Action research, communication, dementia, nursing homes, sensory stimulation

## Abstract

The overall aim of this 2-year Norwegian action research study was to improve the interaction between care workers and patients with dementia in a nursing home by means of sensory stimulation. Furthermore, the aim was to investigate how the staff experienced the interaction with patients suffering from behavioral and psychological symptoms of dementia before, under, and after introduction of sensory stimulation methods in clinical practice. An intervention program consisting of lectures and practical guiding in sensory stimulation was implemented. The care workers participated in group meetings to reflect on the progress. Focus group interviews and participant observations were conducted initially to map exciting practice, and at the end to evaluate potential changes in attitude and skills. Observation notes and interview transcripts were analyzed by means of thematic analysis which revealed a gradual emergence of person-centered care. A phenomenological life-world perspective may serve as a theoretical basis to deepen the understanding of the use of sensory stimulation.

This article relates and discusses the findings from a Norwegian action research study in which health care providers in dementia care tried out a variety of sensory stimulation interventions under supervision. The overall purpose of the study was to improve the interaction between care workers and patients with behavioral and psychological symptoms of dementia (BPSD) in a nursing home by means of sensory stimulation.

Dementia care currently presents a challenge to health care providers on many levels. The number of people suffering from dementia is on the rise worldwide; in 2010, the number was estimated to be 35.6 million (Prince & Jackson, 2009). Recent Norwegian statistics show a prevalence of nearly 70,000 people with dementia, and the numbers are increasing (Engedal & Haugen, 2009). More than half of those diagnosed with dementia live at home. In the later stages of the disease, however, many sufferers become dependent on continuous care, and live out their final years in a nursing home. About 70–80% of the residents in Norwegian nursing homes suffer from dementia in different stages (Engedal, 2000). The care for these patients becomes increasingly challenging as the disease develops and the patients become less able to express themselves in understandable ways. Alternative ways of interacting and building relationships with this group of patients are thus eagerly sought.

Dementia is a degenerative disease. The cognitive decline, memory loss, perceptual problems, and apraxia that result from this degeneration have grave impacts on the everyday life of the person suffering from dementia as well as on his or her surroundings. In its early stages dementia will decrease the person's short-term memory and orientation ability. In the later stages, the disease will also affect verbal communication. Certain BPSD, such as anxiety, sadness, despair, or anger, may appear. These changes will affect the person's ability to cope with and adjust to the environment and his or her interaction with other people. Adjusting the environment to the individual needs of the patients (Zingmark, Norberg, & Sandman, 1993) thus becomes an important issue in dementia care.

When orientation, memory, and the ability to understand reality fail, human relations become increasingly valuable to the patients’ quality of life. The ability of health workers to cooperate with each individual person in accordance with his or her needs and remaining resources is imperative (Wogn-Henriksen, 1997). As verbal communication becomes increasingly difficult, supplementing the care with other non-verbal methods of interaction becomes necessary (Furesund, Lykkeslet, Skrondal, & Wogn-Henriksen, 2007). A variety of non-verbal methods are currently in use, more or less systematically, in the field of dementia care. These methods are related directly to the lived body through various senses, and entail attempts to stimulate the patient and create contact between the care worker and the patient. A sample of terms used for such methods are sensory stimulation, multisensory stimulation (MSS), multisensory environment (MSE), and snoezelen.

Sensory stimulation and MSS refer to a variety of techniques used to stimulate the senses in order to increase alertness and reduce agitation (Gammeltoft, 2005). Sensory stimulation includes auditory, visual, olfactory, tactile, taste, and kinesthetic stimulation (Vozzella, 2007). The notion of snoezelen brings together the ideas of exploring stimuli and being in a state of pleasant relaxation (Baker, Bell, Baker, & Gibson, 2001), and the term was initially used for a room with specialized equipment for stimulating the senses. MSS refers to a process and/or an approach rather than a room (Baker et al., 2001). MSE refers both to a special room much like the snoezelen room (Ball & Haight, 2005), and also to a treatment strategy for persons with dementia living at home (Riley-Doucet, 2009).

The notion of sensory stimulation may be illuminated by theoretical perspectives as different as neuroscience and humanistic philosophy. Back in the 1960s Jean Ayres, an American occupational therapist who worked with children suffering from neurological disorders, developed the theory of sensory integration based on principles from neuroscience, biology, psychology, and education (Scaaf & Miller, 2005). According to Ayres, the sensory integrative processes enable human beings to interact effectively with the environment (Ayres, 1972, p. 1). She accordingly dissociated herself from a traditional view at the time that saw the sensory system in isolation (Spitzer, Smith Roley, Clark, & Parham, 1996).

Snoezelen was developed a decade later by the Dutch therapists Hulsegge and Verheul (1987) while working at a center for people with intellectual disabilities. Although the theoretical underpinnings of this method may be insufficient, the results are interesting. The founders stressed the importance of interpersonal contact and respect for the clients. By attempting to enter into the clients’ world, one may be able to discover or construct “gates” that provide optimal conditions for communication (Hulsegge & Verheul, 1987). Snoezelen has later been referred to as “the enabling approach” and emphasizes the importance of creating an atmosphere of safety and security (Haggar & Hutchinson, 1991). Any theoretical underpinnings related to this view would thus be inspired by humanistic philosophy.

However, according to a phenomenological perspective, in which human beings are regarded as fundamentally embodied (Merleau-Ponty, 1962), both the above mentioned frameworks may be insufficient. According to Kontos, cognitive impairment in people with dementia is often regarded as representing a loss of self-hood (Kontos, 2005; Kontos & Nagile, 2007; Millet, 2011). This view implies a separation of the mind from the body, a dualism that does not take the wisdom of the body into consideration. Hence, one may be in danger of regarding the body as a medium, and sensory stimulation as a substitute for cognition (Kontos, 2005). The body is, however, an active entity constantly aiming at various goals (Carel, 2008). To Kontos (2005) “the body is a fundamental source of self-hood” (p. 553), and she suggests that patients with dementia still perceive the world around them through their senses, through sight, hearing, sense of smell, and tactile sensations. They may have problems arriving at the “correct” interpretation, but they still continue to create a life-world (Wogn-Henriksen, 2012). And they may still try to communicate by means of bodily expressions (Kontos, 2005). Within health care, however, there has traditionally been a tendency to overestimate cognition and forget other sources for the development of human relations. There is currently a growing awareness of alternative ways of handling “inadequate” behavior such as anxiety, sadness, despair, or anger in patients with dementia (Kitwood, 1999). Such behavior is still, however, most often met with medication (Selbæk & Engedal, 2008).

## Sensory stimulation—research

Most studies on sensory stimulation examine the method's effect on different patient groups, with only a few focusing on how the method may contribute to an improvement of the quality of dementia care. Sensory stimulation methods were introduced to improve the patients’ quality of life, and although the purpose of this study was to investigate the care workers’ experience with introducing such methods, any implications for patients were, of course, very important. In the following description of earlier research we have thus found it reasonable to include studies that focus on both health providers and patients.

The use of MSEs for people suffering from dementia increased rapidly in the 1990s. Burns, Cox, and Plant (2000) reviewed the literature in order to investigate both historical and contemporary use. They drew a broad picture of the historical development from the ancient Egyptians’ use of aromatherapy, massage, and baths, up to the present day use of such methods. By reviewing relevant literature the authors classified snoezelen into areas of special education, the care of older people, and other fields such as psychiatry, pain management, and so on. The results in the area *care of older people* showed that the majority of articles indicated positive outcomes, but the studies did not show conclusive results. The authors recommended further research on the topic (Burns et al., 2000).

van Weert et al. published research on the effects on caregivers who used snoezelen (van Weert, Van Dulmen, & Bensing, 2011), and showed that the caregivers succeeded in establishing a more person-centered approach. The caregivers’ affective utterances increased in number and they gained a deeper understanding of the residents’ situations and life values. Their working style became more relaxed; time pressure decreased, they had fewer stress reactions, emotional exhaustion was reduced, and they were more satisfied with their contact with the residents.

Livingston, Johnston, Katona, Platon, and Lyketsos (2005) did a systematic review of 162 studies and measured the outcomes of psychological therapy in managing neuropsychiatric symptoms of dementia; 39 studies focused on psychosocial interventions. Music therapy, snoezelen, and some types of sensory stimulation were reported as being useful during treatments, but showed no long-term effects. Chung and Lai (2009) did a Cochrane review of two controlled trials measuring clinical efficacy of snoezelen or MSS on older people suffering from dementia, and their caregivers. The results revealed no evidence of efficacy of snoezelen on patients’ behaviors, mood, and interaction. Two other reviews measured the effect of snoezelen (Lancioni, Cuvo, & Reilly, 2002; Padilla, 2011) and reported very similar results. In the review of Lancioni et al. (2002), seven studies (of a total 21) were carried out on patients with dementia. Padilla's review addressed the effects of multisensory approaches among other environmental-based interventions. The results from both these reviews—10 years apart—showed some positive effects both during and following the snoezelen sessions. However, the authors stated that the results should be handled with caution due to methodological weaknesses, and that more research is needed.

Finally, we want to mention a qualitative study that may be of relevance to the present study. This study investigated the feasibility and effectiveness of using equipment for MSS sessions in the homes of people with dementia (Riley-Doucet, 2009). Ten family dyads participated. The recommendations stated that sessions should take place when the person with dementia became restless and that the length of the session should be determined by the patient's preferences. The analysis of the data showed positive effects on both the person with dementia and his/her caregiver. The persons with dementia displayed an interest in the equipment, they were more relaxed, and their cognitive state seemed to improve. The caregivers reported experiencing a more relaxed atmosphere, feeling more connected to their loved ones, and the interpersonal relationships improved.

In summary, earlier research gives an unclear picture of the effectiveness of environment-based interventions on people suffering from dementia. However, the quantitative studies seem to report more negative results than the qualitative and the qualitative studies often cover a relatively short time period. We find it interesting that several studies showed an improvement in the relationship between the caregiver and the patient, and studying such interventions over time may consequently yield valuable information.

### Aim

The aim of the present study was to investigate how the care workers experienced the interaction with patients suffering from BPSD before, under, and after introduction of sensory stimulation methods in clinical practice.

## Design, methodology, and method

This study was conducted by an action research design within a phenomenological lifeworld perspective where questions of meanings are essential. Lifeworld is the concrete reality we experience and take for granted in our everyday life (Bengtsson, 2001; Husserl 1936/1970). In our life meaning is often implicit, but can be made explicit through research (Dahlberg, Dahlberg, & Nyström, 2008, p. 36). In addition, a phenomenological view claims that human beings are fundamentally considered relational (Heidegger 1962). According to this view, people are dependent on each other, and create each other's history continuously. As the aim of this study was to improve the interaction between care workers and patients with BPSD, we found it reasonable to choose a relational approach. The phenomenological preposition was of significant value in the data collection. By participant observation, we tried to understand the informants’ experiences. According to van Manen (1990, p. 69) “the best way to enter a person's lifeworld is to participate in it.” Furthermore, the focus group interviews enabled us to get an insight into the informants’ thoughts about themselves and their patients, and thereby explore the meaning of the phenomenon and make it explicit.

In an action-oriented research strategy the researcher intervenes directly in practice instead of assuming an outside position from which to observe what is taking place (Holter & Schwartz-Barcott, 1993; Lewin 1951, 1958; Sjøvoll, 2002). This implies close contact with the participants when seeking to develop collaboration between researchers and practitioners. The social psychologist Kurt Lewin (1951, 1958), who first proposed the term *action research*, wanted to move from mere description of social phenomena to active participation in changing group life through what he called *reflective understanding*. Action research exists in many forms (Kemmis, 2007), with the following common characteristics, according to Holter and Schwartz-Barcott (1993); *collaboration between researchers and practitioners*, *solving practical problems*, *change in practice, and theory development*. In the following, we will describe how these central characteristics were concretized in the current study

Collaboration between researchers and practitioners: The study took place in a nursing home located in a rural area in the middle of Norway. The ward where the study was carried out housed seven patients with dementia. The patient group changed several times during the 2 years, and at the end of the study, only four of the original seven residents were still alive. Six of the eight permanent employees wanted to participate. Four cooperated enthusiastically with the researchers during the whole period, and three temporary workers participated partially. The research team had four members; two researchers from a university college, and two practitioners who were specialized in caring for persons with dementia.

Solving practical problems: The care workers in the nursing home had encountered problems related to communication with persons with BPSD. They were using a variety of communication methods in their work, albeit not systematically. They hoped to improve their communication skills by participating in the current study, and thereby improve their practice.

Change in practice: In order to resolve challenging communication situations and thereby change practice, one of the specialist practitioners supervised the care workers in sensory stimulation methods. Reflection groups were organized to make the practitioners more aware of their attitudes and their possibilities for making changes.

Theory development: The research group did not have ambitions with regard to theory development but hoped to uncover mechanisms that might contribute to an increased understanding and permanent changes of practice.

### Data collection

The study lasted for 2 years and was organized in three phases: “preparing, actions and closing” (Table I). Throughout this process the intervention and the data collection took place simultaneously and are therefore presented together.

**Table I:** Actions and methods during the three phases

Phase 1: Preparing	Phase 2: Actions	Phase 3: Closing
February to June 2010	September 2010 to June 2011	September to December 2011
No action	*Actions:* Teaching Guiding (28 h) Reflection groups (8 meetings)	No action
*Research methods:* Observations (50 h) One focus group interview	*Research method:* Two focus group interviews	*Research methods:* Observations (48 h) One focus group interview

#### Preparatory phase

The aim of this phase of participant observation was to grasp an understanding of the existing communication between patients and care workers ahead of the intervention. During this phase the two researchers who conducted the observations, continuously had brief conversations with the informants about everyday matters. Hence, they learned to know the care workers and their experiences pretty well. Field notes written by each of the two researchers independently were compared and discussed after each observation. At the end of this phase one focus group interview with the care workers was conducted and the whole research group participated. The foci in the interview were related to how the care workers assessed persons with BPSD and what they emphasized in the care of the patients. The preparatory phase lasted for 5 months and amounted to a total of 50 h.

#### Action phase

The aim of this phase was to make the care workers able to cope with challenging situations by using sensory stimulation and to investigate their experiences of the intervention. The specialized practitioner emphasized a phenomenological view of human beings by underlining the significance of embodiment and human relationship. She gave lectures on dementia, BPSD, and different sensory stimulation methods. In addition, she guided the care workers through challenging everyday situations for a total of 32 h. Methods used were hand and foot massage, music therapy, physical activities, and muscle-joint compression. The caregivers tried out a variety of stimulation tools adapted for persons with dementia.

A reflection group was established to further supervise, discuss, and evaluate the progress related to the implementation of the methods. Eight reflection meetings took place during this 10-month phase. Two focus group interviews, one before, and one after the intervention, were conducted to document how the caregivers experienced the intervention process.

#### Closing phase

The aim of the last phase was to grasp possible changes in the care workers’ experiences and how they felt able to implement the new methods. Participant observation of a total of 48 h followed the same model as described in phase one. This 6-month phase was completed with one last focus group interview.

### Analysis

Guided by a phenomenological openness we tried to grasp the meaning of the informants’ experiences. Furthermore, the analysis was influenced by thematic analysis with reference to Braun and Clark (2006).

The researchers typed the observation notes and a secretary transcribed the interviews verbatim. The four members of the research group participated in the analysis and had several 2-day meetings over a period of 1 year.

The members of the group first stated an intention of gaining an overall understanding of the whole material, both interviews and field notes. Then, each researcher analyzed the focus group interviews separately. The field notes contributed to contextualize the analysis of the interviews. Each researcher pondered upon the whole data to obtain preliminary themes, which then was subject of discussion in the whole group. We searched for final themes by asking questions such as: What did the informants tell us about their daily challenges?, How did they use sensory stimulation methods in communicating with the patients?, and How did they experience their own role in using these methods? During this process we gradually agreed upon the meaning of these experiences and named the final themes.

### Ethical considerations

Participation was voluntary and the care workers signed an informed consent form in advance. They were informed about the study and their right to withdraw at any time without any consequences. The staff nurse informed the patients about the researchers’ “visits” in the ward. She considered in each case whether the patient himself/herself was able to consent or whether it was sufficient to ask the relatives to consent on his/her behalf. All relatives gave informed consent in writing on behalf of the patients, whereas the patients gave their oral consent in situations where the head nurse found it necessary. Patients suffering from dementia are vulnerable and in need of protection. An ethical dilemma might arise from the fact that the patients may not remember the information given a few minutes ago, and the researchers had to pay close attention to any possible signs that the patients were becoming uncomfortable and withdraw when necessary. The Regional Committee for Medical and Health Research Ethics approved the study (2009/2260-7).

## Findings

Analyses of the data revealed that the participating care workers experienced going through a process of change in their attitude towards the patients. They increasingly recognized the importance of developing relationships in human interaction. In spite of this, a complete cultural change did not seem to take place. According to the informants’ experiences the changes emerged gradually through a combination of supervision in practice, continuous reflection, and acquisition of knowledge. Two main themes appeared through the analysis.

### Gradually viewing symptoms as meaningful expressions

At the start of the preparatory phase the care workers explained what they understood by challenging behavior and how they responded to such behavior. Challenging behaviors were regarded as symptoms and may consist of physical attacks, such as hitting, spitting, and pinching. One of the informants described her own reaction to the behavior of a female patient:

I do not think she always knows that she pinches us … then we try to get away … we pretend that nothing has happened and her behavior is a great challenge to us, because her inhibitions against hitting and kicking are very low.

Patients who just wander around or repeatedly say that they want to go home, may also constitute a challenge. The caregivers regarded such behavior as a symptom with spillover effect. *When one patient wants to go home, others also want to go home …* Another gave an example of a patient who wandered around all day long.

All the time she wants to go someplace, but does not know where. She puts on a lot of clothes and walks around … if I tell her the truth about where she is, she becomes irritated, resigned, or offended.

The care workers also found it challenging that patients ask the same thing over and over throughout the day; one woman kept asking all day whether she still had any children.

During the course of the first year of the study the staff explained that they increasingly searched for meaning in the patients’ behavior and thereby gradually seemed to change their attitude. They claimed that they had learned to see peculiar behavior more as a result of a challenging situation than as a symptom of a difficult patient. One of the care workers described a patient who did not want to eat her food:

She refuses to open her mouth, so it is impossible to feed her. Any new caregiver who helps her will often fail. When I assist her, I always start by touching her hand, holding her hand and then she gradually starts to eat.

She added that this patient might have misunderstood the situation and therefore did not trust the caregiver, who had to spend some time building trust. *You cannot go straight to the task, she needs some preparation to understand what is going on and what she has to do*. By observing the patient's reactions, the care provider understood that the patient needed time to prepare. She needed to understand that the situation was a meal and that the care worker wished her well. Another informant told about how she tried to understand patients with poor verbal language: *We try to read their body language. Perhaps she is in pain … sometimes she can tell us … other times we get no answer*. Sometimes they saw the behavior as a response to a critical situation: *Because he is vulnerable he becomes angry*.

The observation notes confirmed changes in the informants’ attitude and caring activities. We registered that when the caregivers experienced a situation as challenging, they more frequently started to reflect on the patients’ needs.

### Gradually realizing the importance of human relationships

At the beginning of the observation period we noticed that the caregivers organized the day according to certain routines. In the morning the staff gathered the patients in the dining room for singing, playing cards, telling stories, and other activities. They tried to get most of the patients to participate in these activities.

Five patients are sitting in the dining room. One of the caregivers has brought songbooks and helps the patients find some well-known songs. Some patients seem to love singing. Those who are not able to use words are humming. Most of them remember many parts of the songs. This was a nice hour with small talk in between the singing (field note).

At other times the care workers brought up a topic from the old days. They tried to get everybody involved in the conversation, and many patients seemed to like this activity. We did sometimes observe, however, that one or two of the patients became angry and said that they did not want to sing or they withdrew from the conversation.

The interviews uncovered a gradual realization that sensory stimulation required individual adaptation and that human relationships were a precondition for succeeding. The caregivers talked about their wish to care for the patients in a qualified way. They wanted to care for each patient in the best possible way, *treat each one in a special way, give all of them what they need*. They talked about sensory stimulation as a toolbox with tools to use in different situations: *Sensory stimulation is an alternative to pills. Sometimes it works, sometimes it doesn't, just like pills*. And another caregiver added:

Sensory stimulation is a new possibility, if we do not manage to establish contact during massage and use of oil, we try with music and songs. We have to reach each patient and therefore we have to use different methods.

They explained how they tried out various methods for each patient, and they used the word “improvisation” and explained how they proceeded.

The way I see it … you cannot just carry out a method, you do it to establish contact in order to create a good feeling and perhaps make the patient more easily accessible for contact with others.

Many of the caregivers emphasized that they felt they were using themselves in a new way, they were much more aware of how they used their hands when touching the patients. They also found it important to prepare the patients before introducing various kinds of sensory stimulation. At the end of the project many of the patients enjoyed foot massage before they went to sleep at night. The caregivers reflected much more on the situations and their own participation in the relationship. The patients’ behavior was understood more as a relational phenomenon than as individual actions.

## Discussion

The overall purpose of the present study was to improve the interaction between care workers and persons with BPSD. The aim was to investigate how the staff experienced the interaction with the patients, before, under, and after the introduction of sensory stimulation methods in clinical practice.

The findings indicate a change in attitude among the care workers during the 2 years the project lasted. The most obvious changes were perhaps related to the development of a new understanding of the individual suffering from dementia. Gradually, the care workers saw each patient emerge as an individual with different needs that required different approaches. Although the care workers initially tried to offer sensory stimulation methods that might be applied to all the patients, they gradually started to focus on activities that were adapted to each individual patient. The persons with dementia were to a greater extent regarded as individuals, and not as a general patient with general symptoms and behavior to be met with general interventions.

In line with Kitwood's (2003) ideas, the care workers became more person-oriented. Kitwood presents a paradigm where the individual comes first, a view he contrasts to a standard paradigm built on medical knowledge of disease and symptoms. According to this, the reference should no longer be a person-with-DEMENTIA, but a PERSON-with-dementia (p. 17). The care workers in the current study changed focus from general symptoms of dementia to the person who suffers from the illness. This is in line with other research (Cox, Burns, & Savage, 2004; Minner, Hoffstetter, Casey, & Jones, 2004; van Weert et al., 2004). Other studies point out that sensory stimulation leads to a changed relation between the care worker and the patient. In studies where the caregivers were present during the sensory stimulation sessions, they describe the relationship as more relaxed and with a higher degree of equality and trust between patient and caregiver (Cox et al., 2004; Minner et al., 2004; van Weert, van Dulmen, van Spreeuwenberg, Bensing, & Ribbe, 2005b; van Weert, van Dulmen, van Spreeuwenberg, Ribbe, & Bensing, 2005a). It may thus not necessarily be the method that matters, but rather the unique relationship that evolves between the patient and the caregiver (Furesund et al., 2007).

Although a person-oriented perspective is obviously valuable and important within dementia care, we will question whether this is sufficient for acquiring the necessary insights into what it means to suffer from dementia. The present study indicates that the care workers changed their attitude to patients with BPSD. They increasingly started to ponder upon the meaning of the patients’ behavior. Could an outburst from a patient be an expression of pain or disappointment rather than of anger or aggression? Could the sudden behavior be rooted in the misunderstanding of an utterance from a fellow patient or a health care provider?

A phenomenological lifeworld perspective may contribute to a possible understanding of another human being's universe of meaning. Humans seek meaning, and encounters between people are always charged with meaning (Bengtsson, 2001; Husserl 1936/1970). This does not mean that we always understand each other; misunderstandings are quite common because of different pre-understandings (Gadamer, 1960/1995). The greater the historical, cultural, or social distance, the greater the chance of misunderstandings. It may be difficult for a person with good health to understand the ailments of old age. Similarly, it may be even harder for a mentally healthy person to understand persons who are suffering from reduced cognition, as in the case of dementia. But reduced cognition and impaired memory do not mean that a person no longer searches for meaning. In lifeworld phenomenology, human beings are fundamentally considered relational; people are dependent on each other and continuously create each other's history (Heidegger, 1962). It may thus be reasonable to claim that health care providers in a nursing home are co-creators of the patients’ universe of meaning. Accordingly, we are responsible for creating each other's personhood, a view that is not very clearly illuminated in Kitwood's writings (Baldwin & Capstick, 2007). Misinterpretation and misunderstandings from both parties may contribute to isolating the person with dementia in his/her own universe if his reactions are regarded solely as symptoms. The woman who did not open her mouth during meals may be an example of this. The care worker may have proceeded too fast and the patient did not understand the situation, that this was a meal. Closeness and touch from the care worker prior to the meal seemed to be a language the woman could understand and the feeding went well.

In nursing homes it is well known that patients suffering from dementia often get ready to go home, to their childhood home, even though they have not lived there since they were children. Memory fails them and the time perspective is distorted. Nevertheless, this is their perspective on reality. Being prevented from carrying out one's intentions in such situations may cause both anxiety and anger. However, in some situations, it seemed as if the patients calmed down when being met with different kinds of sensory stimulation such as music in combination with hand or foot massage. Rejection, on the contrary, often led to aggression, which again might spread to the other residents.

The two examples mentioned above illustrate that the human lifeworld is constituted by some basic structures; we are embodied, we live in time and space, and we are dependent on human relations (Heidegger, 1962; van Manen, 1990). In illness and aging these structures may change, and this causes changes in our lifeworld. In patients with dementia such changes may appear as reduced cognition and reduced memory. In a distorted time perspective the past may take over for the present and both fellow man and space appear as unfamiliar. The care providers in the present study did not know phenomenological lifeworld perspective, but the lectures, reflections, and concrete training over time turned into new knowledge and a good foundation for changed understanding. Supplementing the person-oriented paradigm in dementia care with a lifeworld-centered paradigm, which is in line with Kontos (2005), seems reasonable. Hence, we will claim that lifeworld phenomenology may be regarded as a theoretical foundation for sensory stimulation methods.

## Methodological considerations

Compared to former qualitative studies on intervention with sensory stimulations in dementia care, our study covers a much longer period of time, which may be regarded as a strength. Our findings are also in line with earlier results showing an improved relationship between patients and caregivers (Riley-Doucet 2009; Van Weert et al., 2011). A weakness in our study, however, is that not the entire staff wanted to participate in the project. This may have prevented a complete cultural change. In action research it may be important to ensure that the whole staff participates. As mentioned in the introduction, one of the characteristics of action research is collaboration between researchers and practitioners for the sake of making change. Even though not all the staff took part in the study, the intervention aroused great interest among the care workers who indicated that they had learned a lot.

## Conclusion

According to the participants in the present study, sensory stimulation may improve the quality of dementia care by expanding the care workers’ repertoire of potential methods for making contact with patients suffering from BPSD. Sensory stimulation seems to provide opportunities for accommodating the individual person's needs and thereby establish a person-centered perspective. An expansion of a person-centered approach with a phenomenological lifeworld perspective may form a theoretical basis for, and a deeper understanding of, the patients’ universe of meaning.
